# Supplementary material for: Revisiting silk: a lens-free optical physical unclonable function
Source: Nat Commun. 2022 Jan 11;13:247. doi: 10.1038/s41467-021-27278-5 (PMC8752800; doi:10.1038/s41467-021-27278-5)
Supplement: Supplementary file 1 — Supplementary Information [file 41467_2021_27278_MOESM1_ESM.pdf]

Supplementary Information

Revisiting silk: a lens-free optical physical unclonable function

Min Seok Kim[1], Gil Ju Lee[1,2], Jung Woo Leem[3], Seungho Choi[4], Young L. Kim[3,5]*, and Young Min Song[1,6,7]*

[1]*School of Electrical Engineering and Computer Science (EECS), Gwangju Institute of Science and Technology, 123, Cheomdangwagi-ro, Buk-gu, Gwangju, Republic of Korea, 61005*
[2]*Department of Electronics Engineering, Pusan National University, 2 Busandaehakro 63 beon-gil, Geumjeong-gu, Busan 46241, Republic of Korea*
[3]*Weldon School of Biomedical Engineering, Purdue University, West Lafayette, Indiana 47907, United States*
[4]*Department of Biomedical Engineering, Yonsei University, Wonju 220-710, Republic of Korea*
[5]*Purdue Quantum Science and Engineering Institute, West Lafayette, Indiana 47907, United States*
[6]*Anti-Viral Research Center, Gwangju Institute of Science and Technology (GIST), 123, Cheomdangwagi-ro, Bukgu, Gwangju, Republic of Korea, 61005*
[7]*AI Graduate School, Gwangju Institute of Science and Technology (GIST), 123, Cheomdangwagi-ro, Bukgu, Gwangju, Republic of Korea, 61005*

*Correspondence to: ymsong@gist.ac.kr, youngkim@purdue.edu

These authors contributed equally: Min Seok Kim, Gil Ju Lee

Supplementary Notes

Supplementary Note 1. Optical simulations for a single fiber

A two-dimensional beam propagation method (BPM) built in a commercial software (RSoft, Synopsys, USA) was used to investigate the self-focusing phenomena of a single fiber. A grid size of 5 nm was used in the simulation to produce numerically stable results. A plane wave with a 645 nm-wavelength was used as the light source. The boundary conditions were the transparent boundary conditions. An absorption coefficient of 0.02 was applied to the fiber media shown in Fig. 2a to realize the opaque fiber. A programmable language (MATLAB, Mathworks, USA) was exploited to generate the simulation domains with different nanohole densities of 0, 5, and 10%. The domain for the random hole was defined as a 25000 × 25000 blank matrix. One element of the matrix was present at 1 nm. Thus, the dimensions of the blank matrix were 25 µm × 25 µm. The diameters of all the nanoholes were set to 25 nm. The generated holes, which had 25 diameter elements, were located in the domain with arbitrary center positions. The located holes were recorded as '1' in the matrix. To check the density of the hole, the ratios of 1 and 0 were calculated for every generation. In addition, we used feedback to avoid the overlapping of each nanohole (Supplementary Fig. 25). Supplementary Fig. 3 shows one of the generated single fibers with nanofibrillar structures. The refractive indices of the background, microfiber, and nanoholes were 1, 1.6, and 1, respectively.

Supplementary Note 2. Theoretical analyses of the fiber bundle for PUF applications

Three-dimensional BPM simulations were performed using commercial software (RSoft, Synopsys, USA) to analyze the lens-free imaging features in the fiber bundle geometries. A commercial programmable language (MATLAB, Mathworks, USA) was used to produce random fiber media with three densities: 70, 80, and 90%. The domain for the random fiber was defined as a 1000 × 1000 blank matrix. One element of the matrix was 1 μm. The overall size of the fiber bundle domain was fixed at 1×1 mm^2. The fiber bundles consisted of 30 μm-wide fibers. The virtual rectangular fiber, which had 30 elements for width and an infinite length, was defined to fill the domain. The virtual fiber was generated in a domain with an arbitrary center position and angle. The located fiber was recorded as '1' in the matrix. To check the density of the fibrous medium, ratios of 1 and 0 were calculated for every generation. The boundary conditions were the transparent boundary conditions. To validate the statistical approach, we repeated the generation of a random fibrous medium. All of the generated fiber bundle media are shown in Supplementary Fig. 6. The generation method for a fiber bundle is shown in Supplementary Fig. 26. The geometrical parameters (*i.e.*, average diameters and number of holes) in each medium were analyzed. To calculate the average diameter of the medium, the diameter of the circle was calculated by dividing the total hole area by the number. The geometrical statistical results confirmed the consistency of the generated fiber bundle media. To simplify the computation, the refractive index of all fibers was set to 1.6, and all fibers were assumed to be opaque by introducing an absorption coefficient of 0.02.

Image simulations were conducted to confirm the unique responses from randomly generated fibrous media (Supplementary Fig. 7). Three light sources (*i.e.*, red, green, and blue) were launched from three incident angles, i.e., −15°, 0°, and 15°, for CRPs. Without a cut-off process, the simulated images did not exhibit remarkably focused spots. The cut-off process

provided apparent and unique focal spots at far distances, such as 0.8 and 1.0 mm, in the red, green, and blue images. Based on this result, colored lights from different incident angles could serve as true random seeds for PUF applications. The colored images in Fig. 1d were also produced using this simulation.

Supplementary Note 3. Measurement setup for the self-focusing effect

Supplementary Fig. 4 exhibits the optical image of the measurement setup for observing the self-focusing feature of native silks under an incoherent light source. Three solid-state LEDs (LED625L, LED525L, LED470L, Thorlabs, Inc., Germany) produced red, green, and blue light, and a native silk fabric was mounted between the glass slides to ensure the flatness of the silk fabric. A 1x objective lens (*i.e.*, relay lens; 30 mm focal length f/4, Thorlabs, Inc., Germany) transferred the focal spots generated by the silk fabric to a commercial image sensor (IMX226, Sony, Japan). The image sensor was a chromatic sensor that separated colored lights. To capture the focal spots using silk depending on the distance, a single-axis motorized stage (CMA-25PP, Newport, USA) was used, which was controlled by a motion controller (ESP-300, Newport, USA). The images were obtained with a movement step of 10 μm. The obtained results are shown in Figs. 2g and 2h.

Supplementary Note 4. Conversion method of the response to bitstream

MATLAB (Mathworks, Inc., USA) was used for image processing and bit extraction. The captured images had a three-color space composed of red, green, and blue. For image processing, the image was decomposed using three channel images. Next, each image was exploited to generate bitmaps. To equalize the LED illumination, the original and blurred images were subtracted from each other through a Gaussian filter. Moreover, a binning process was performed to reduce the peak/edge noise (Supplementary Fig. 23). An image size of 2048 × 2048 was binned to an image size of 32 × 32 pixels. A threshold was applied to the binned image for digitization of the data. Finally, von Neumann debiasing was conducted in each bit column stream (*i.e.*, 32 × 1 bits). Von Neumann debiasing follows the following rule. (i) If a pair of bits in sequence were 00 or 11, the pair of bits moved to the 2nd pass extractor. (ii) If the pair of bits were 01 or 10, the bit pair moved to a debiased bit sequence. (iii). Reconsidering the bits in the 2nd pass extractor, the bit pairs were again grouped into pairs. (iv) If the first and second pairs were different (i.e., 0011 or 1100), the bit pairs in the 2nd pass extractor were maintained (Supplementary Fig. 24). If the number of debiased column bits was more than four bits, the first four bits were extracted. Otherwise, the processing moved to the adjacent bit column sequence and extracted four bits. This process was repeated until 64 bits were collected.

Material	PUF Type	Measurement Device	Reference
**T cells**	Optical (organic)	4× lens camera	[1]
**TiO_2, ZnO**	Optical (inorganic)	15× lens camera	[2]
**Quantum dots**	Optical (organic)	200× lens camera	[3]
**Au nanoparticles**	Optical (metallic)	40× lens camera	[4]
**Thermoplastic materials**	Optical (organic)	2.8× lens camera	[5]

Supplementary Table 1. Summary of various optical PUF devices in terms of their materials and objective lens specifications. All reported optical PUFs adopted lens elements to distinguish random seeds in the PUF response.

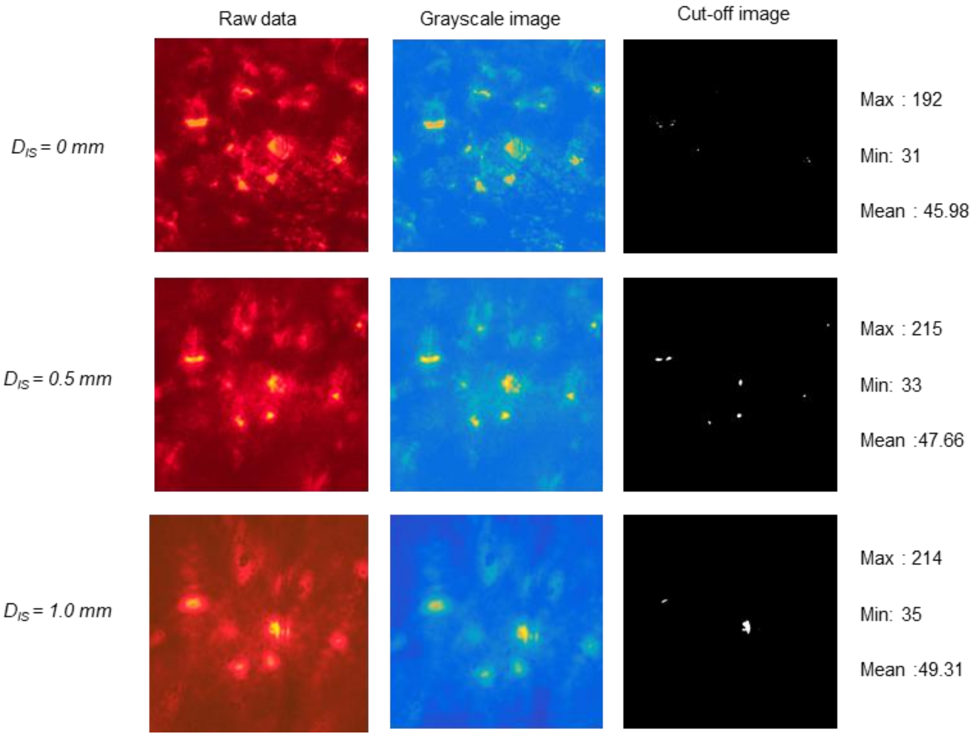

Supplementary Fig. 1 | Bright peaks of various Z position Raw data, grayscale images, and cut-off images for each image. Maximum, minimum, and mean values of each image are noted.

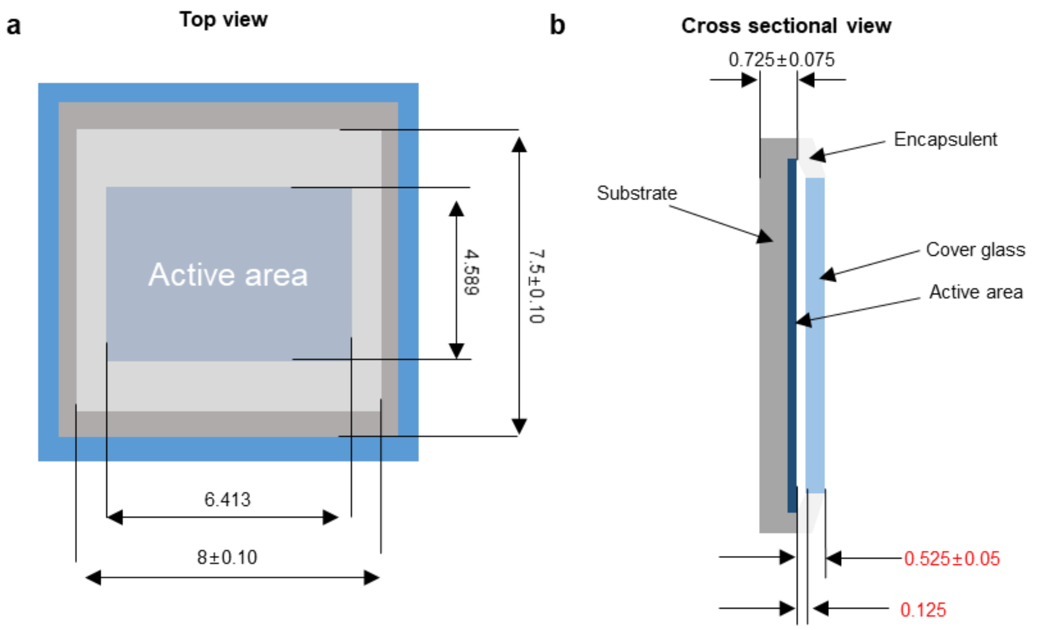

Supplementary Fig. 2 | Image sensor for LOP-PUF. Schematic illustration of the image sensor (MT9J003, On semiconductor, USA). (a) The top view of used image sensor. (b)The cross sectional view of used image sensor. The unit is millimeters. Lens-free imaging requires a sufficient focal length to form focal spots on the active area through the cover glass. The total thickness of the air gap and cover glass was 0.6–0.7 mm.

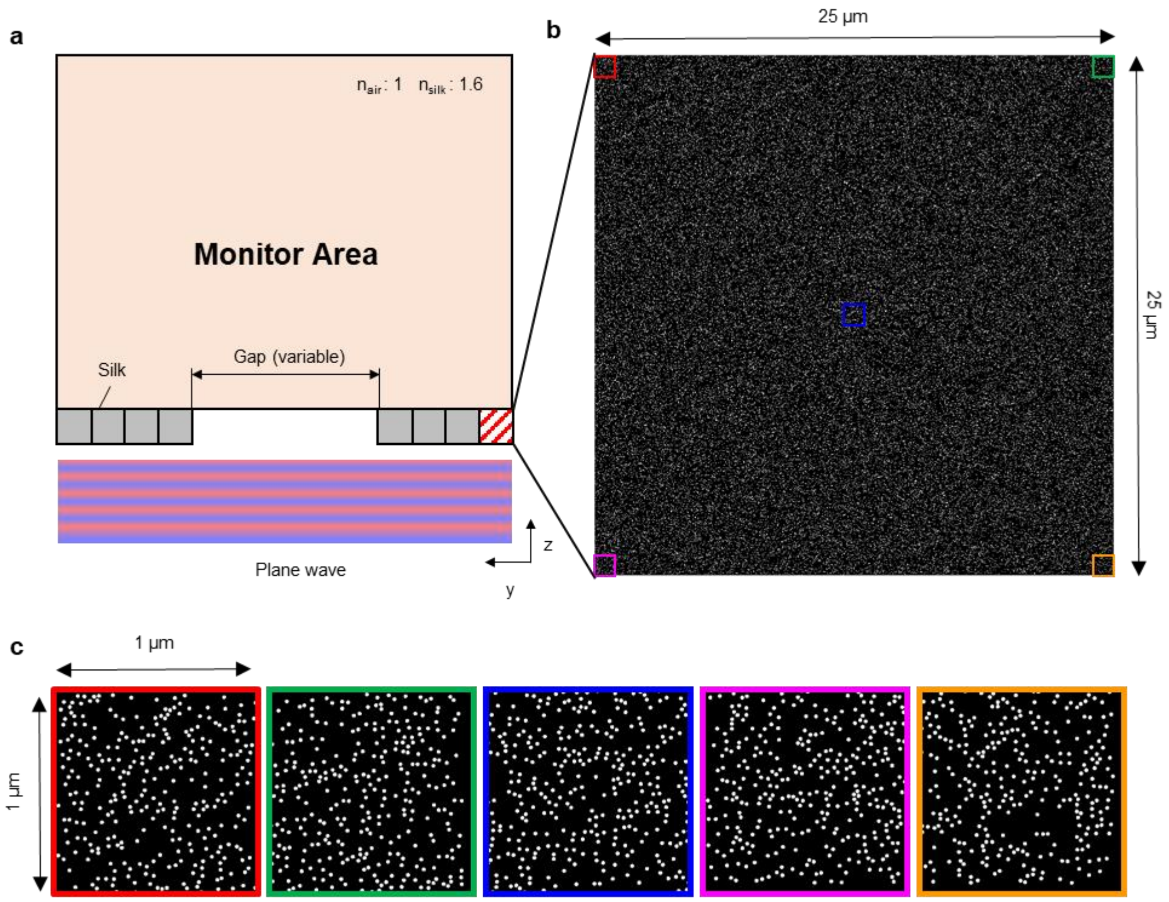

Supplementary Fig. 3 | Detailed model of optical simulation (a) Schematic of the simulation domain. The silk medium, which was 25 μm-thick, was located at the zero of the z position. The monitor domain was 200 μm-wide and 5 mm-long. Various gap values were applied for parametric analysis. (b) The refractive index profile of the random hole medium at a density of 10 %. (c) The magnified images at the designated areas in (b).

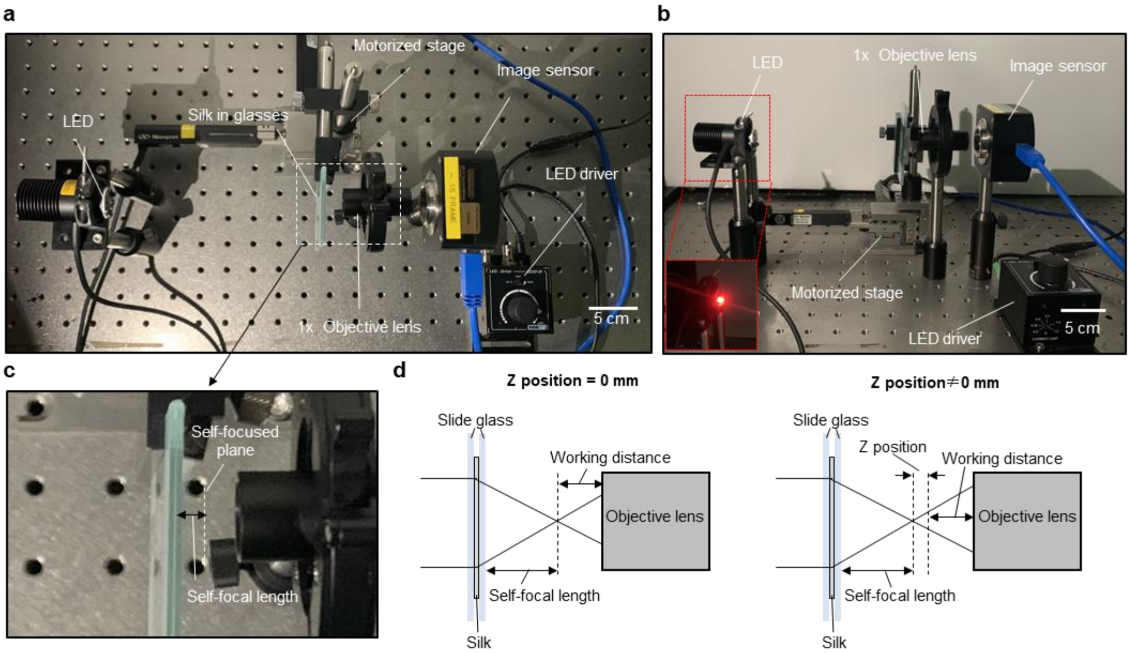

Supplementary Fig.4 | Measurement setup for Z position dependence. (a, b) Photographs of the experimental setup for the self-focusing effect. The motorized stage was used for detecting the Fraunhofer region. The 1x objective lens had the same effect as the direct contact of silk on the image sensor. (c) The magnified image of the objective lens and silk was embedded in the glass. (d) Schematic illustration of the '*Z* position'. At $Z = 0$, the self-focal length of the silk matched the working distance of the objective lens. Otherwise, *Z* was not zero.

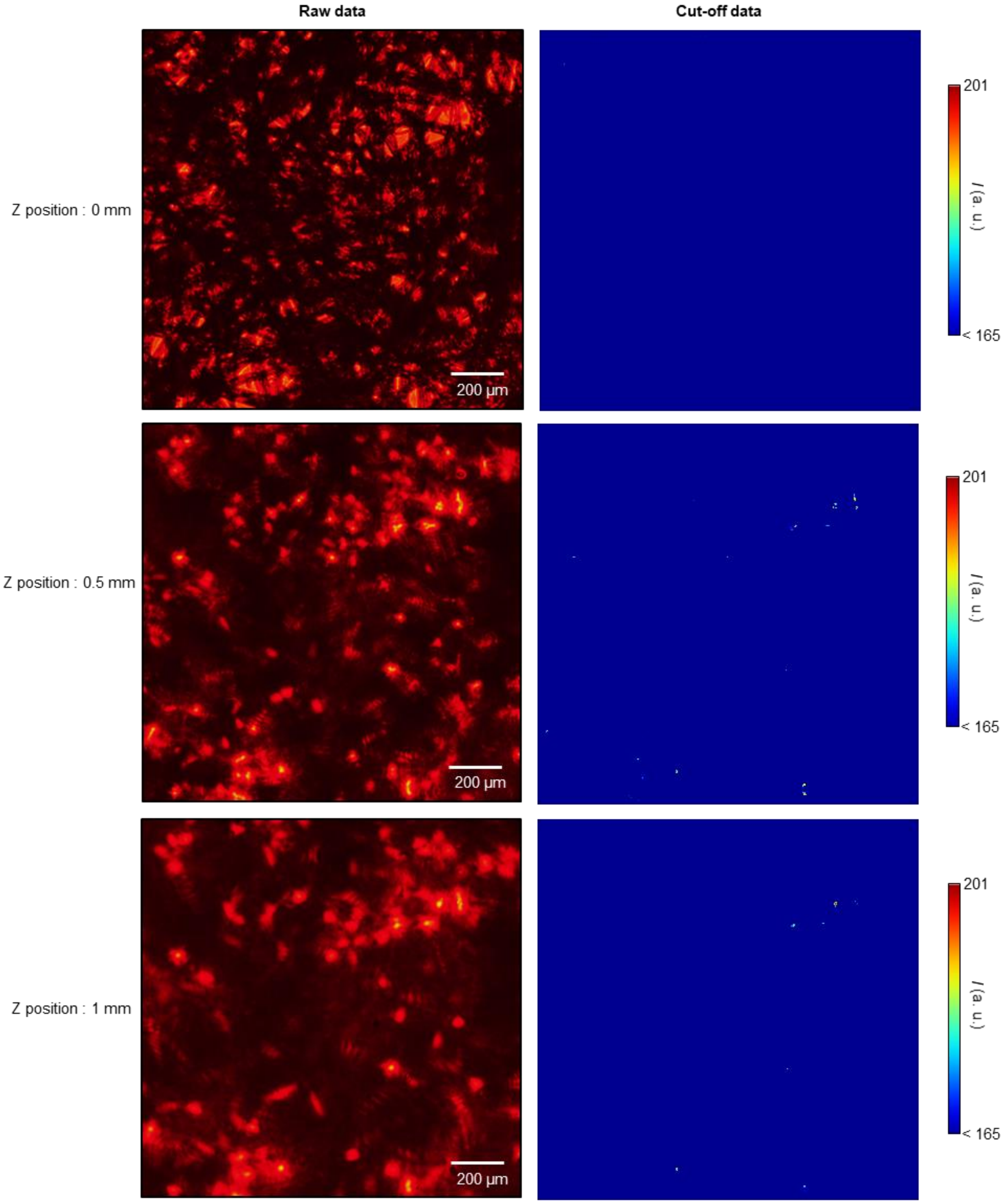

Supplementary Fig. 5 | Obtained image at three Z position. The obtained images and cut-off images under red LED light for three different *Z* positions. The cut-off value was 80 % of the maximum intensity value, *i.e.*, 201.

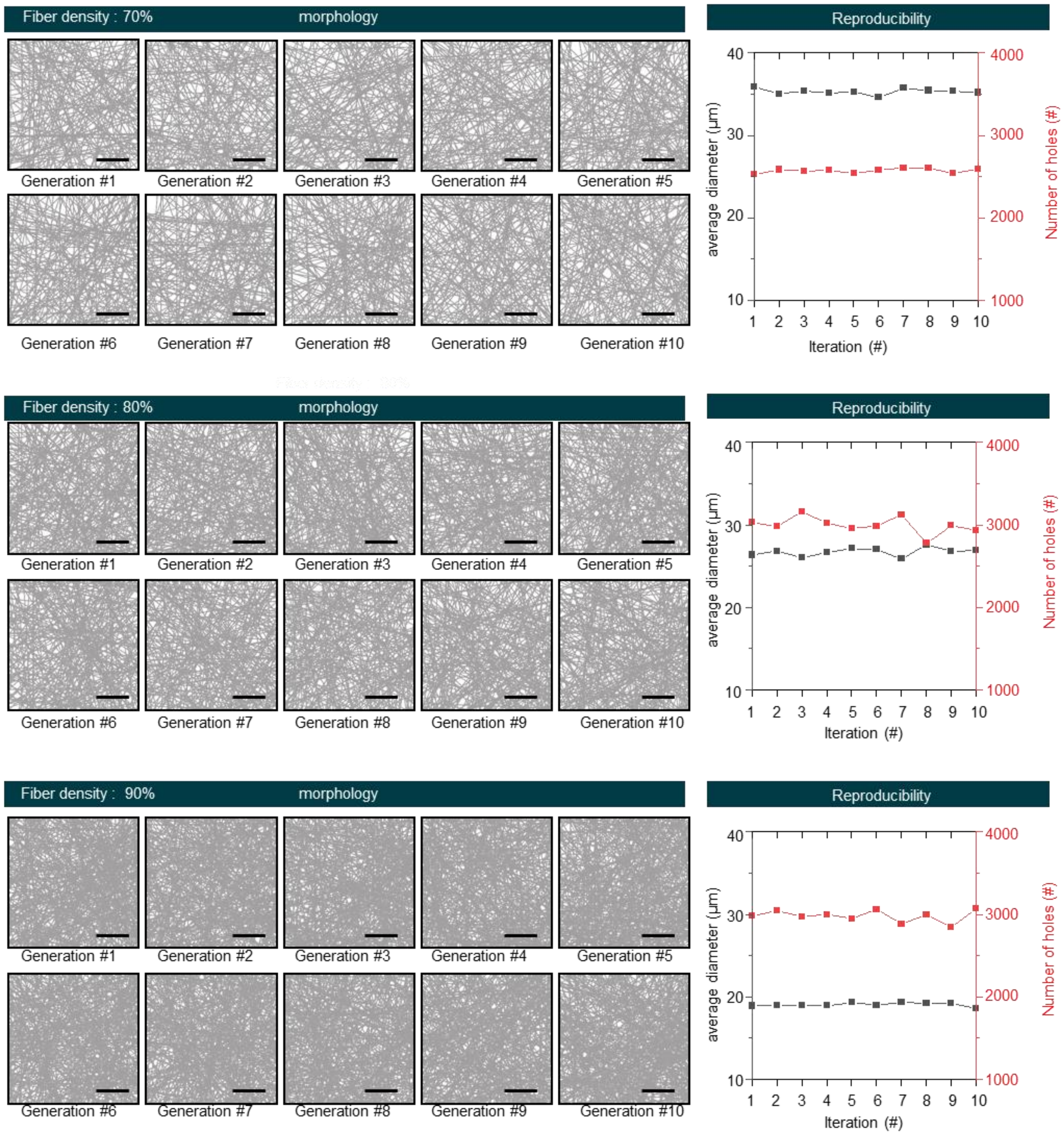

Supplementary Fig. 6 | Mophologies and reproducibility of the virtually generated fibrous media. The mophologies of the virtually generated fibrous media of three densities (70 %, 80 %, and 90 %) for 10 random generations. Each morphology of fibrous media had different structures. However, at the same density, the fibrous media had similar average diameters and numbers of holes. The scale bar is 1 mm.

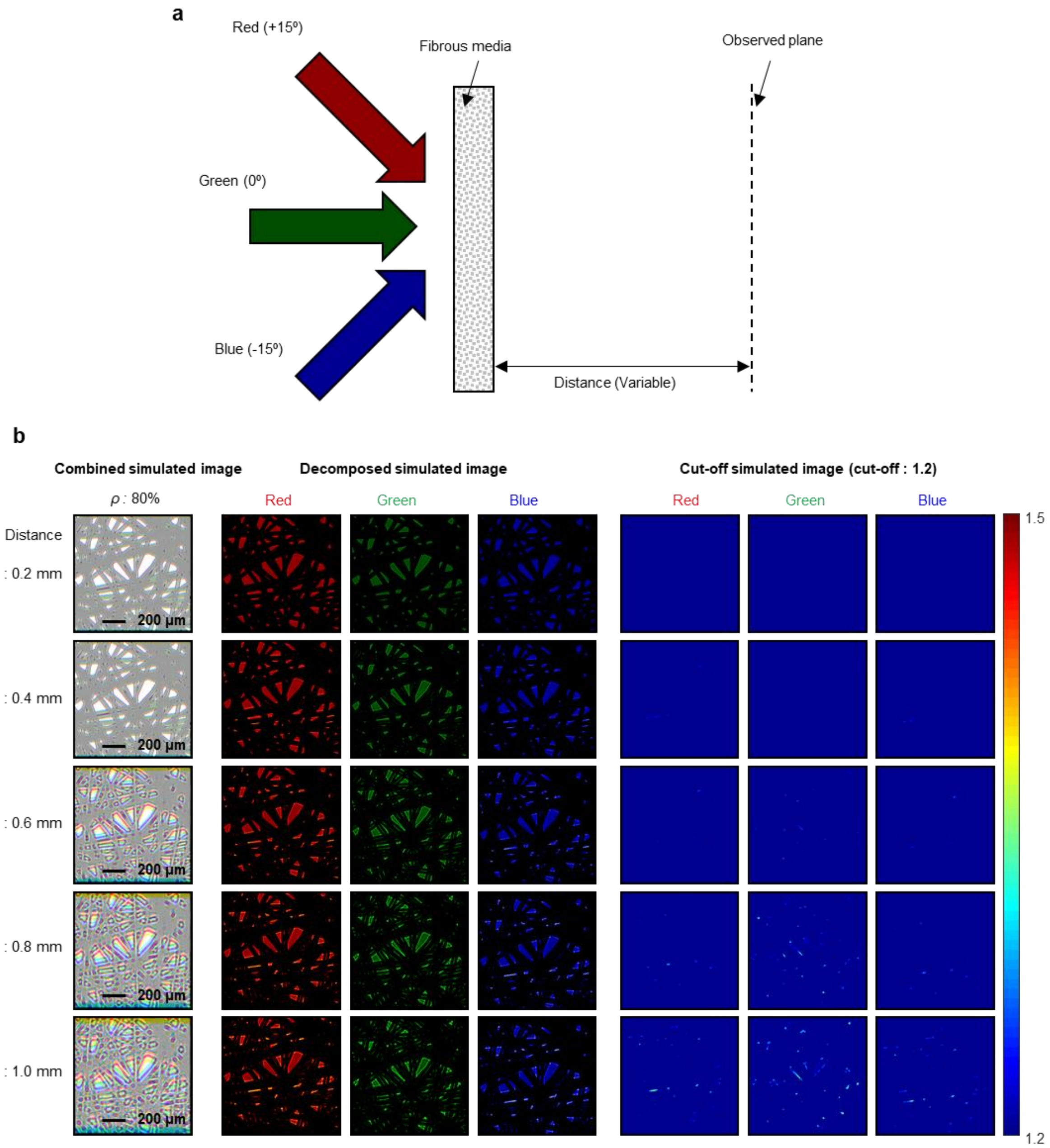

Supplementary Fig. 7 | Optical simulation of fibrous medium. (a) Optical simulations for a density of 80 % with three different wavelengths, i.e., 635, 530, and 435 nm, corresponding to red, green, and blue. The three plane waves were launched at angles of −15, 0, and 15° to the fibrous medium. (b) The cut-off images display the generation of strong focal spots within the Fraunhofer region. For fibers of 80 % density, which had many candidate holes with diameters of 27 μm and 48 μm, peak points started to appear from 0.4 mm.

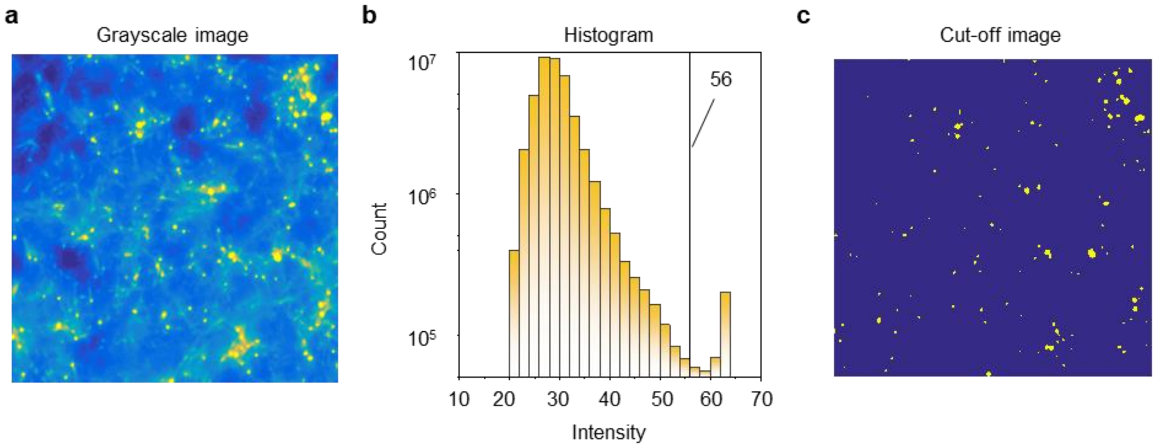

Supplementary Fig. 8 | Threshold for image process. (a) Grayscle image of raw data obtained using the LOP-PUF. (b) Histogram of the grayscale image which shows a dip at ~80% of the intensity range. (c) Binary image obtained using 80% of threshold.

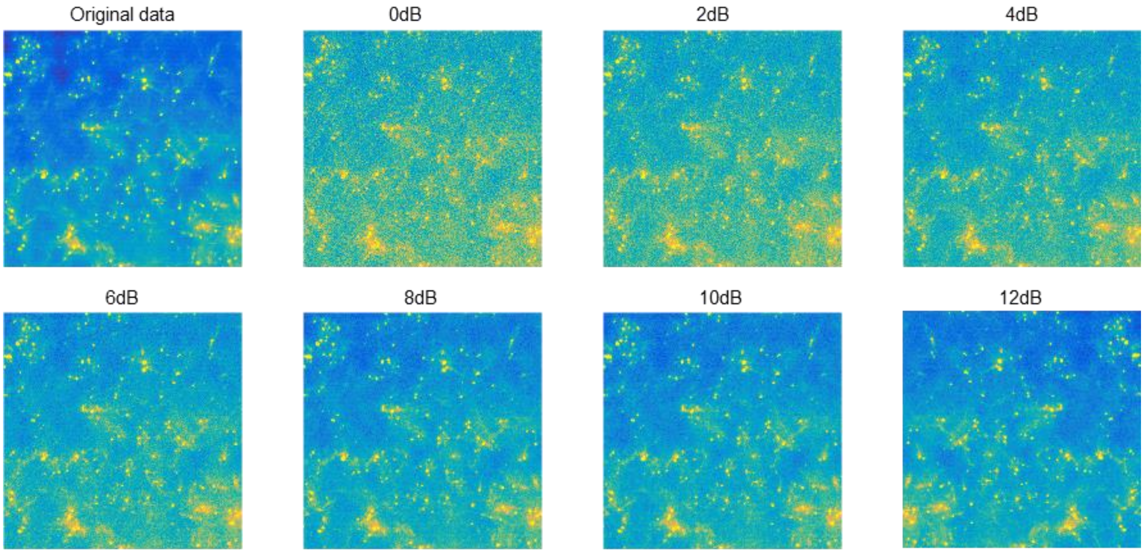

Supplementary Fig. 9 | Effect of various white noise on raw data. The obtained raw data with the LOP-PUF system and artificially noise added images with different SNRs (*i.e.*, 0, 2, 4, 6, 8, 10, and 12 dB).

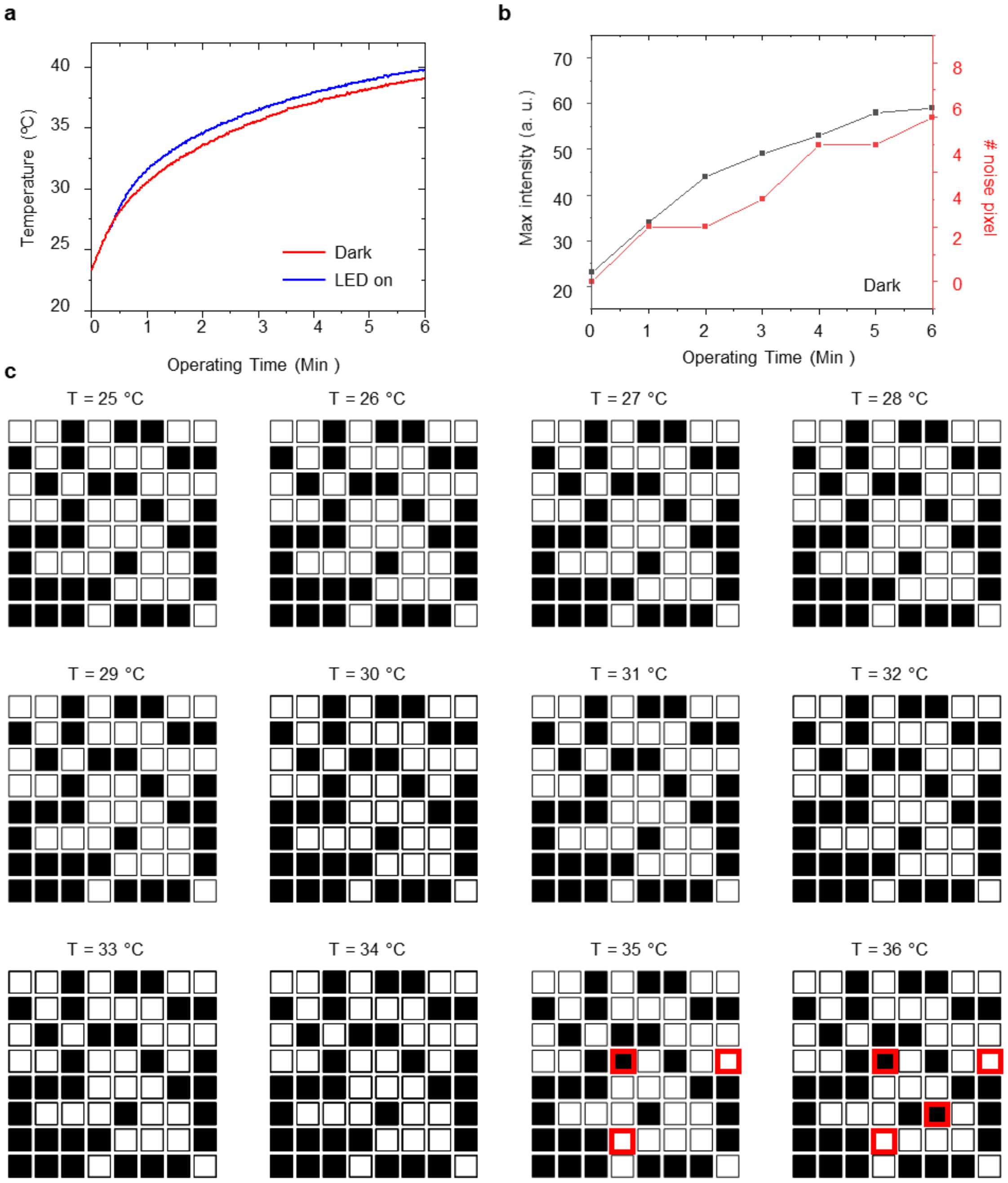

Supplementary Fig. 10 | Temperature effect of bit extraction. (a) Temperature variation of an image sensor as a function of the operating time of a LOP-PUF module with and without under the blue light illumination with the center wavelength of 467 nm (optical intensity of 64 μW/cm^2). (b) Maximum noise intensity (black solid line) and number of thermal noise (red solid line) as a function of the operating time of a LOP-PUF module in dark. (c) Bit maps extracted from a LOP-PUF module at various temperatures of 25-36 ºC.

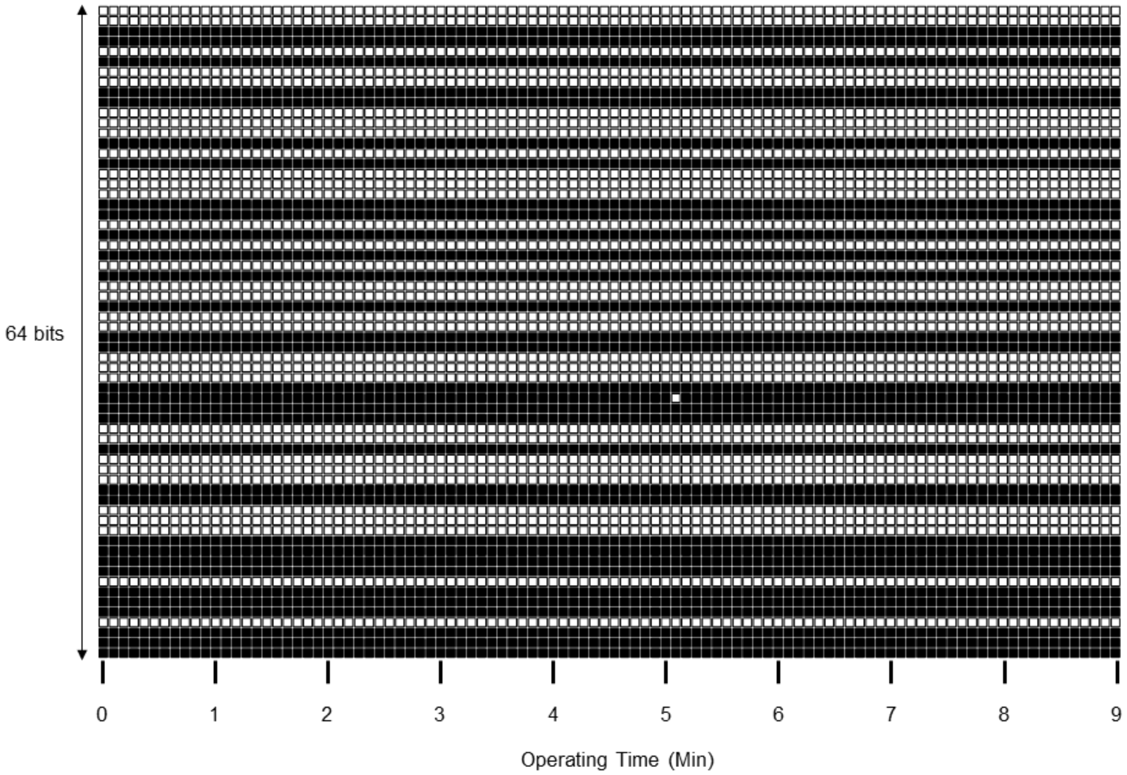

Supplementary Fig. 11 | Extracted bit with temperature control. Bit sequences obtained by a LOP-PUF module with the temperature control (*i.e.*, 27 °C).

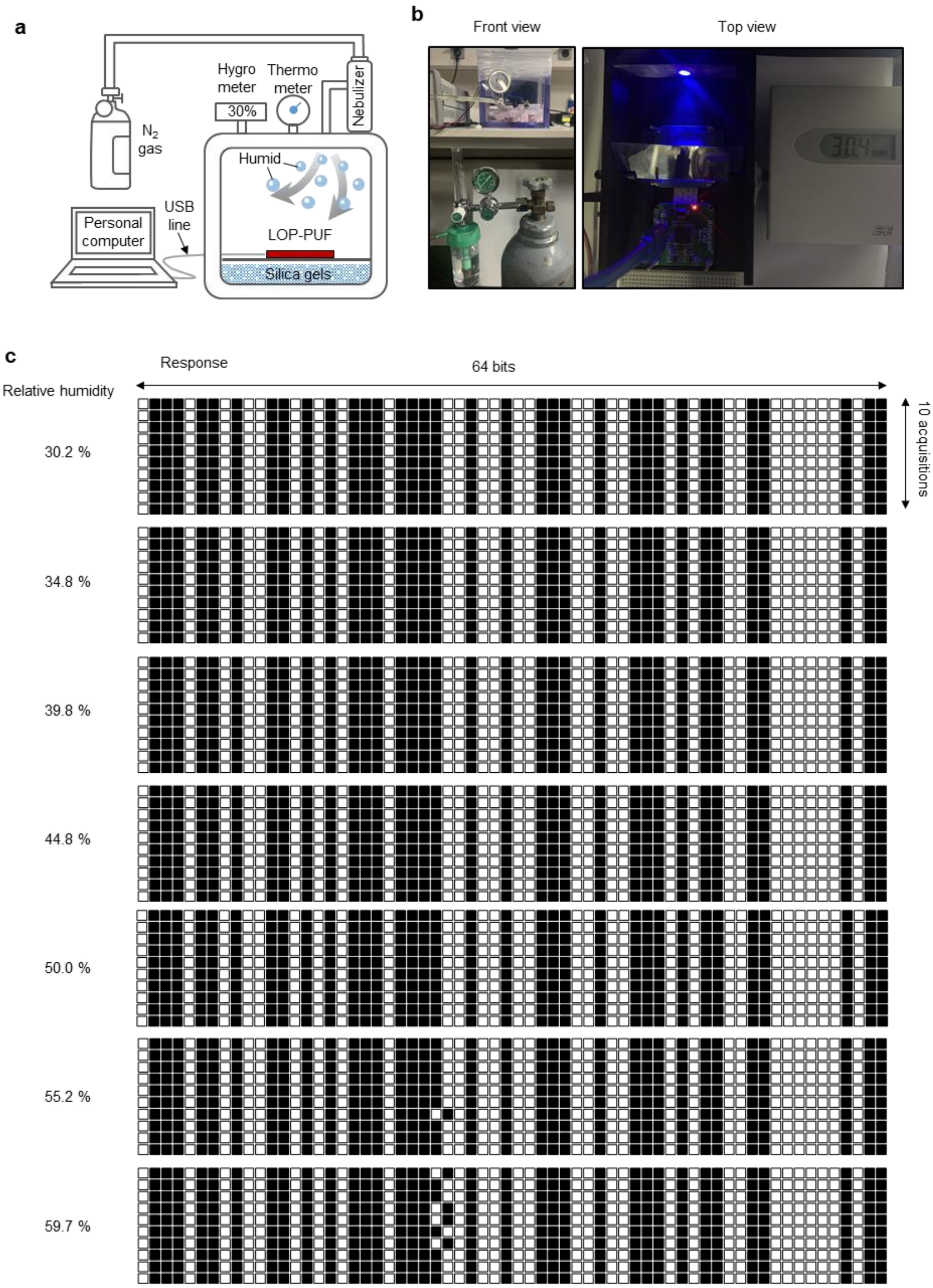

Supplementary Fig. 12 | Humidity effect of bit extraction. (a, b) The schematic (a) and photograph (b) of measurement setup for various humidity values. Silica gels were used to lower the initial humidity, and the humidifier raised the humidity. (c) The obtained responses at each relative humidity value under the blue light illumination with the center wavelength of 467 nm (*i.e.*, RH = 30.2, 34.8, 39.8, 44.8, 50.0, 55.2, and 59.7 %).

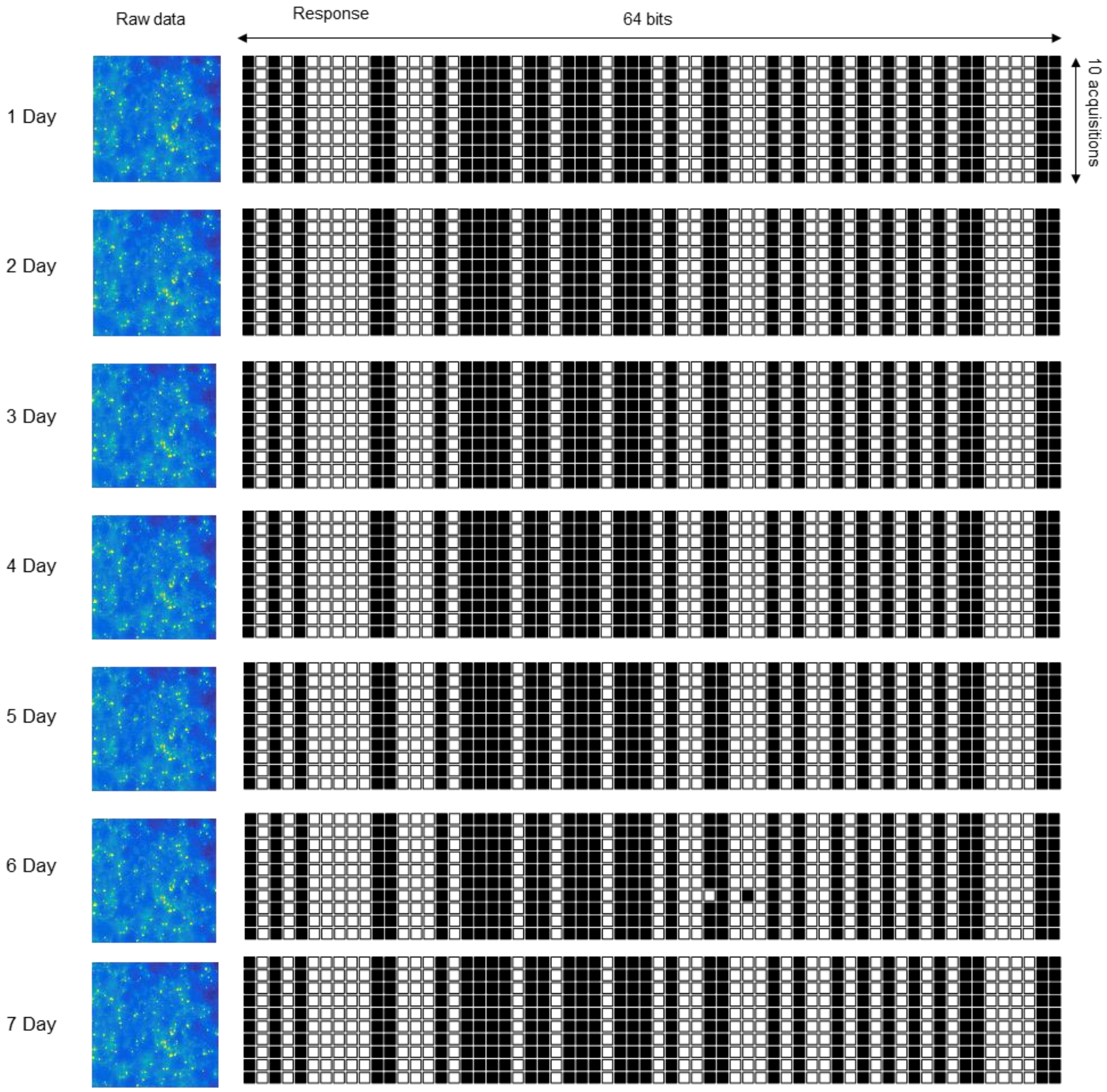

Supplementary Fig. 13 | Measurement of long-term stability. Raw data (left) and bit response (right) obtained with the LOP-PUF over seven days. The bit responses show '1' bit error on the sixth day in the measurement conducted over a seven-day period. Data acquisition was repeated 10 times per measurement under the blue light illumination with the center wavelength of 467 nm.

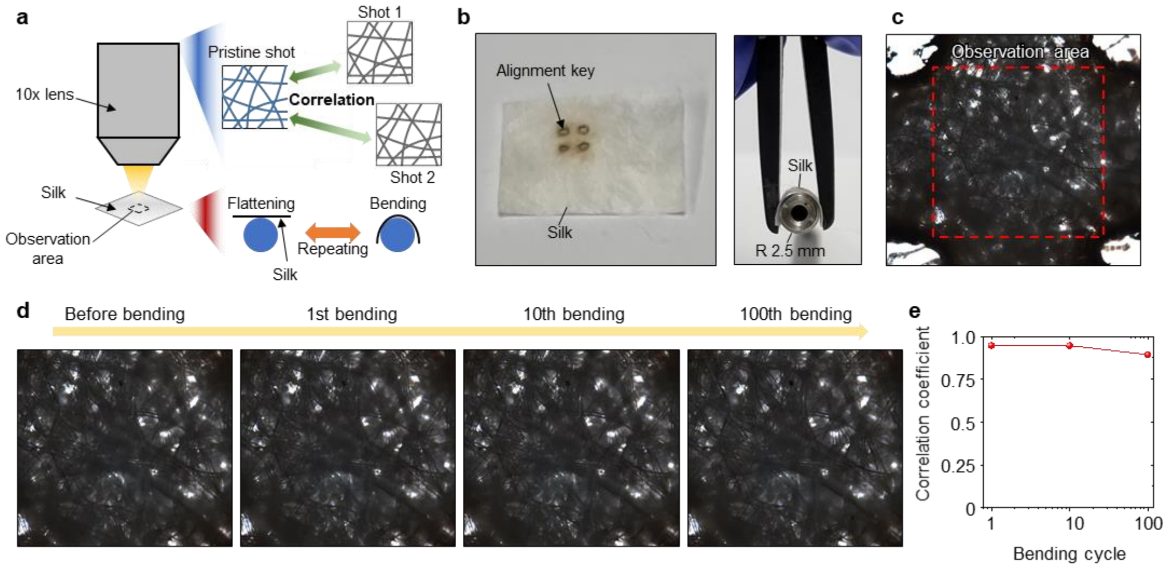

Supplementary Fig. 14 | Measurement of Mechnical stability. (a) Schematic illustration of measurement setup for bending test. (b) Photographs of observed silk with alignment keys (left) and bent status of silk with the radius of curvature of 2.5 mm (right). (c) Observed silk by 10x objective lens. Red dashed box indicates that the observation area. (d) Raw images with different bending cycles (*i.e.*, before bending, 1st, 10th, and 100th bending). (e) Correlation coefficients of raw images with bendings compared with the raw image before bending.

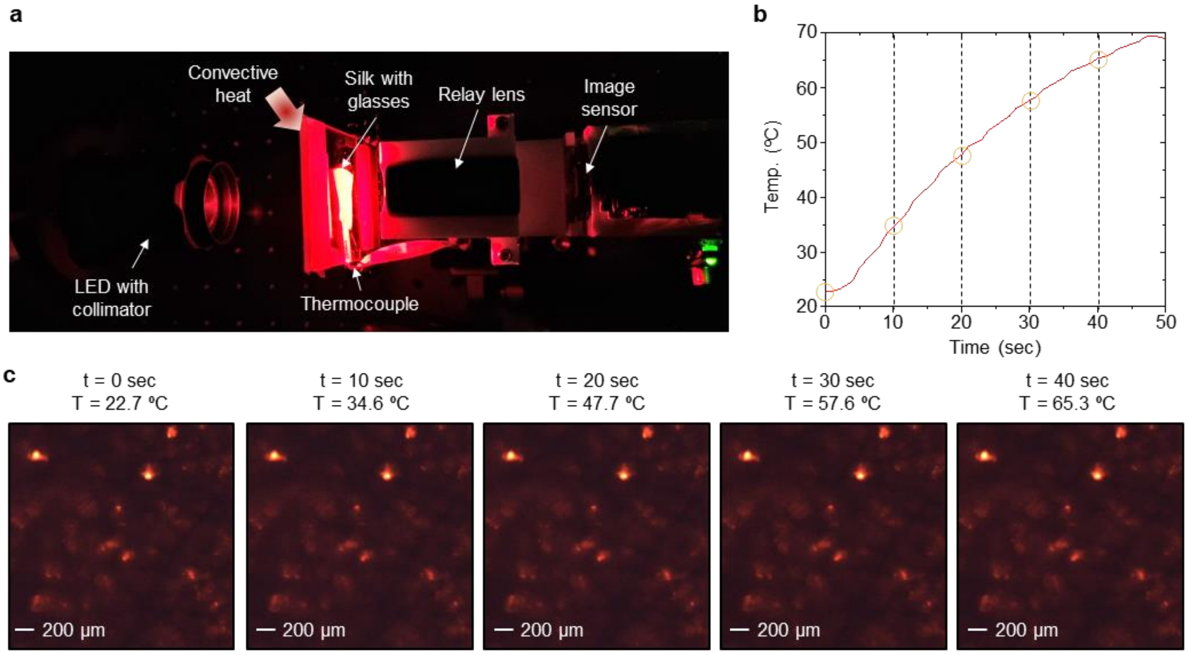

Supplementary Fig. 15 | Thermal stability measurement of silk (a) Photograph of measurement setup for self-focusing effect under heating. (b) Temperature of silk material. A convective heating was used to raise the temperature. (c) Captured raw images with the time interval of 10 sec. The temperature of silk was increased from 22.7 to 65.3 °C.

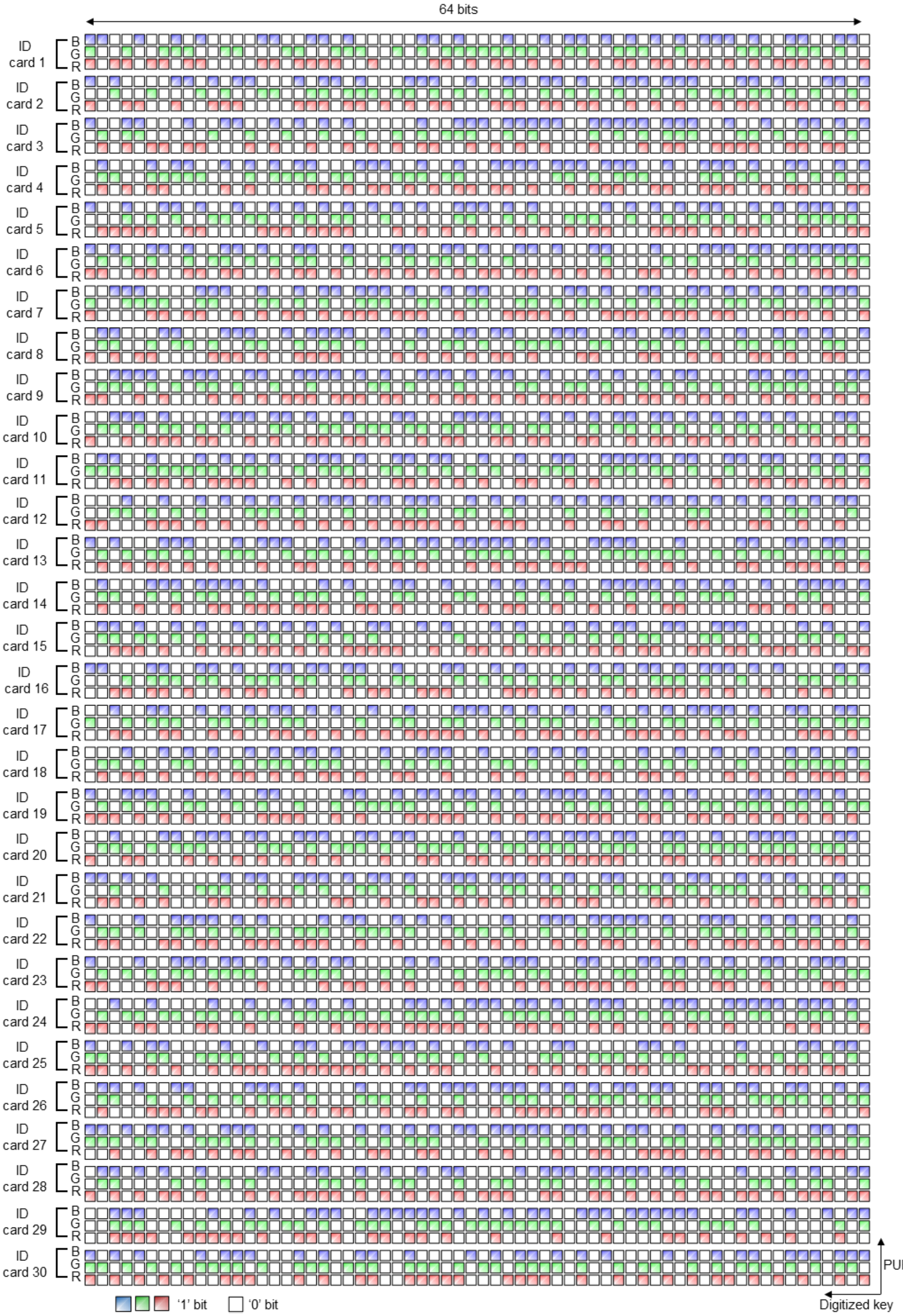

Supplementary Fig. 16 | Bit sequences from 30 different silk ID cards. The bit sequences from 30 different silk ID cards obtained by the LOP-PUF for three spectrally separate LEDs. The three LEDs were positioned at an angle in the LOP-PUF to illuminate the light obliquely.

a

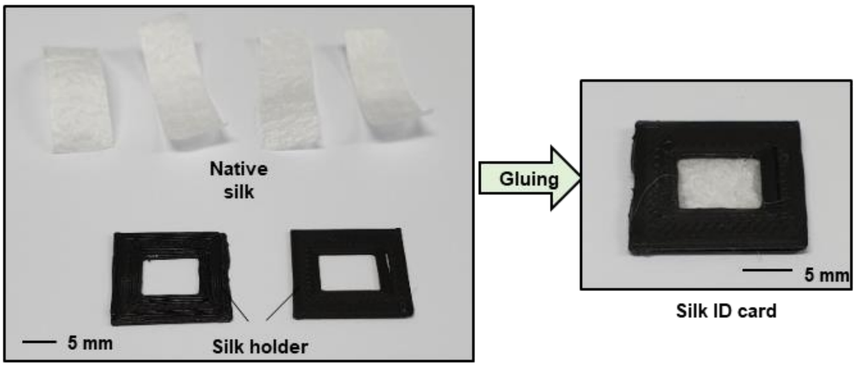

b

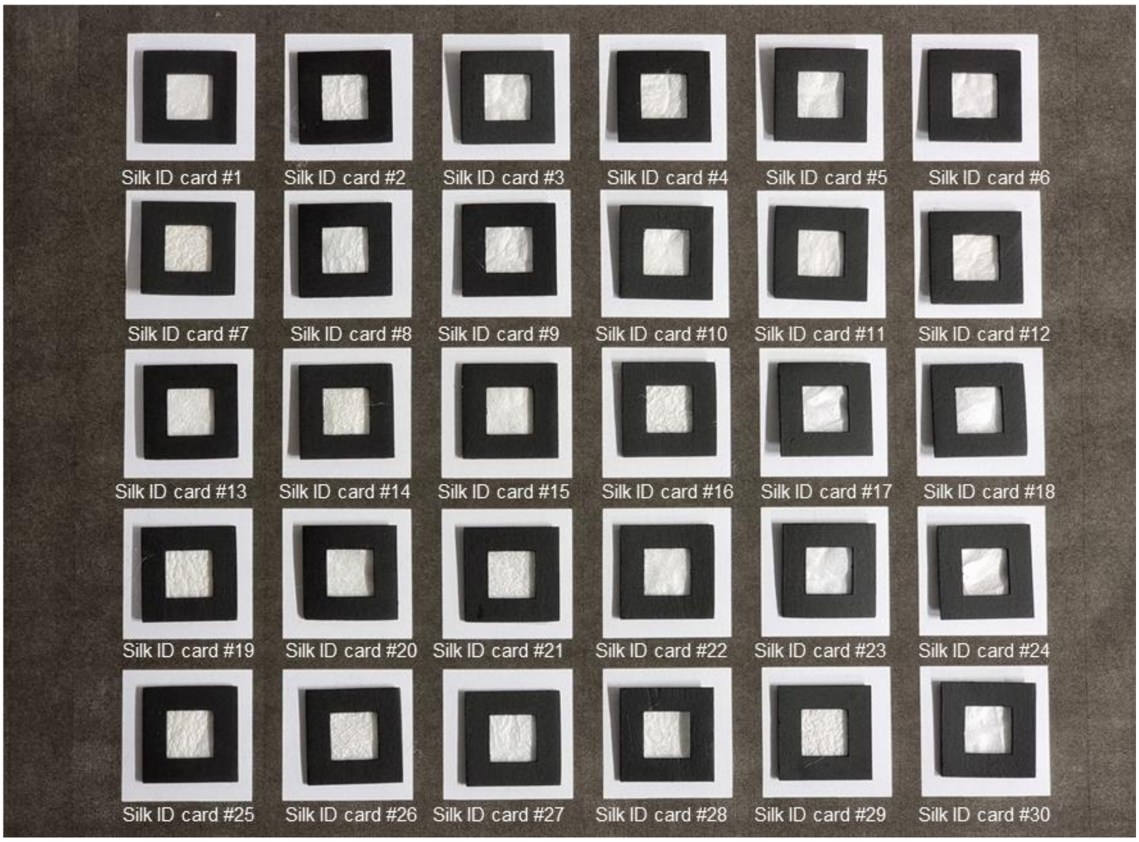

Supplementary Fig. 17 | Fabrication process of silk ID cards (a) The fabrication process for the silk ID cards. (b) Photography of the silk cards for bit extraction with the LOP-PUF module.

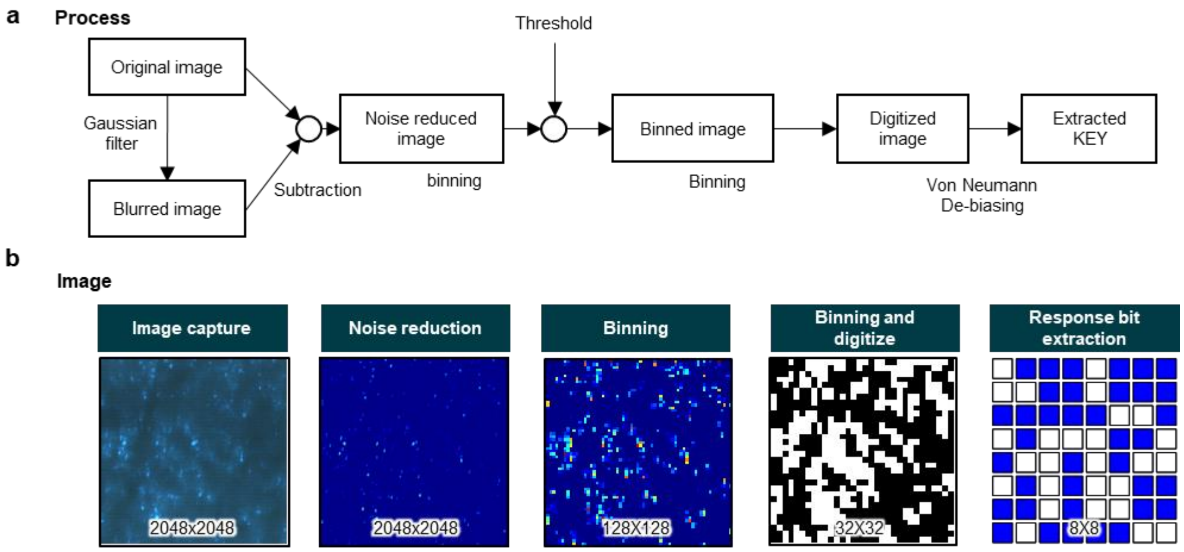

Supplementary Fig. 18 | Data processing flow chart. (a) Detailed data processing flow chart for bit extraction. (b) Representative data formats for each step of the bit extration process for the LOP-PUF.

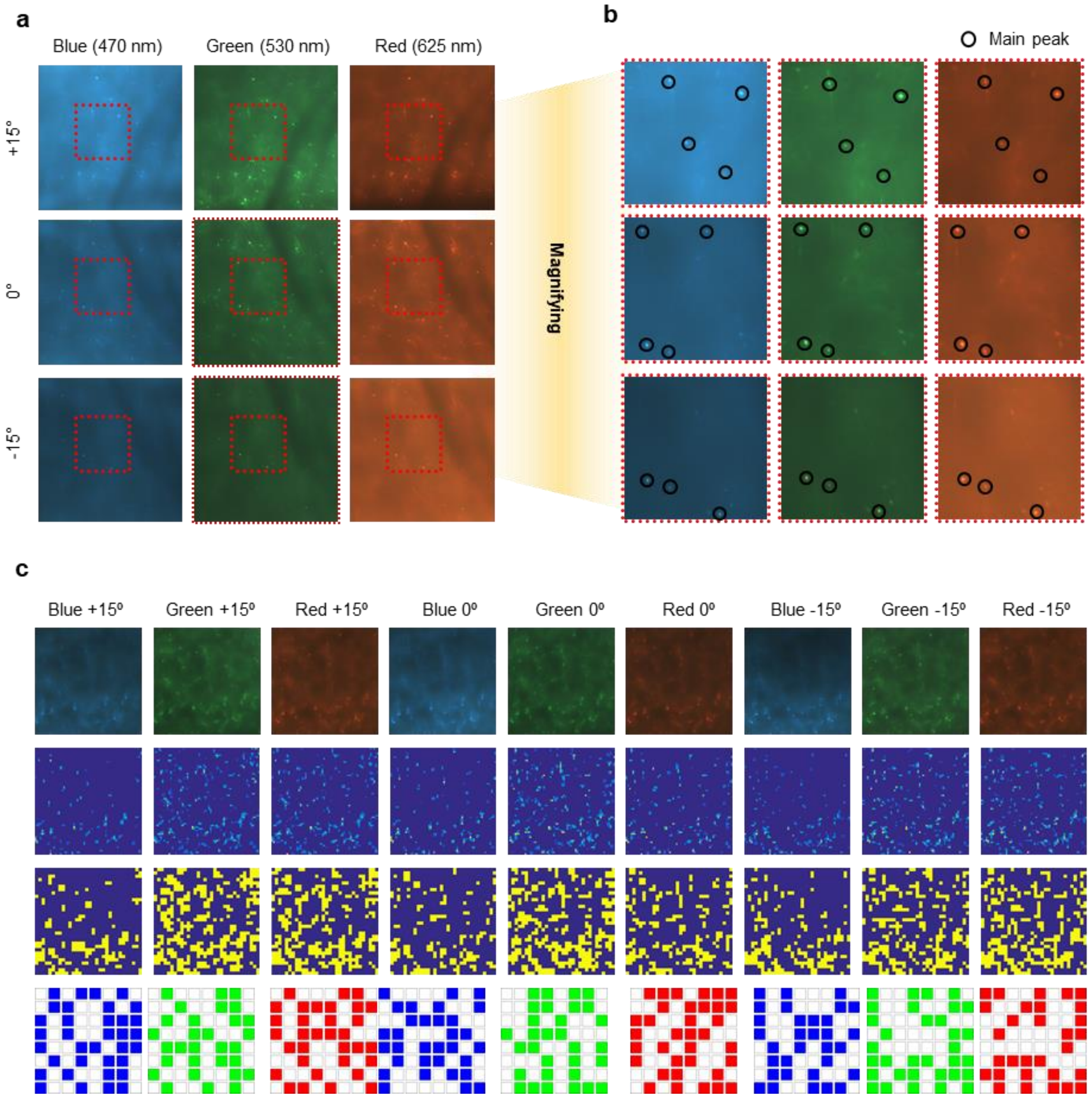

Supplementary Fig. 19 | Peak position change according to the angle of incidence of light (a) The obtained raw data with the silk ID card at different wavelengths with different incident light angles. (b) The magnified images of the obtained images indicated by the red boxes in Fig. S8. The peak position of the raw data was maintained at same incident light angle. In the case of varying incident light propagation, the position of the peak point changed. (c) The obtained raw data with native silk and the data processing for each response. Each challenge was created with a combination of spectral multiplexing (*i.e.*, 467 nm, 525 nm, and 637 nm) and different incident angles (i.e., +15º, 0º, −15º).

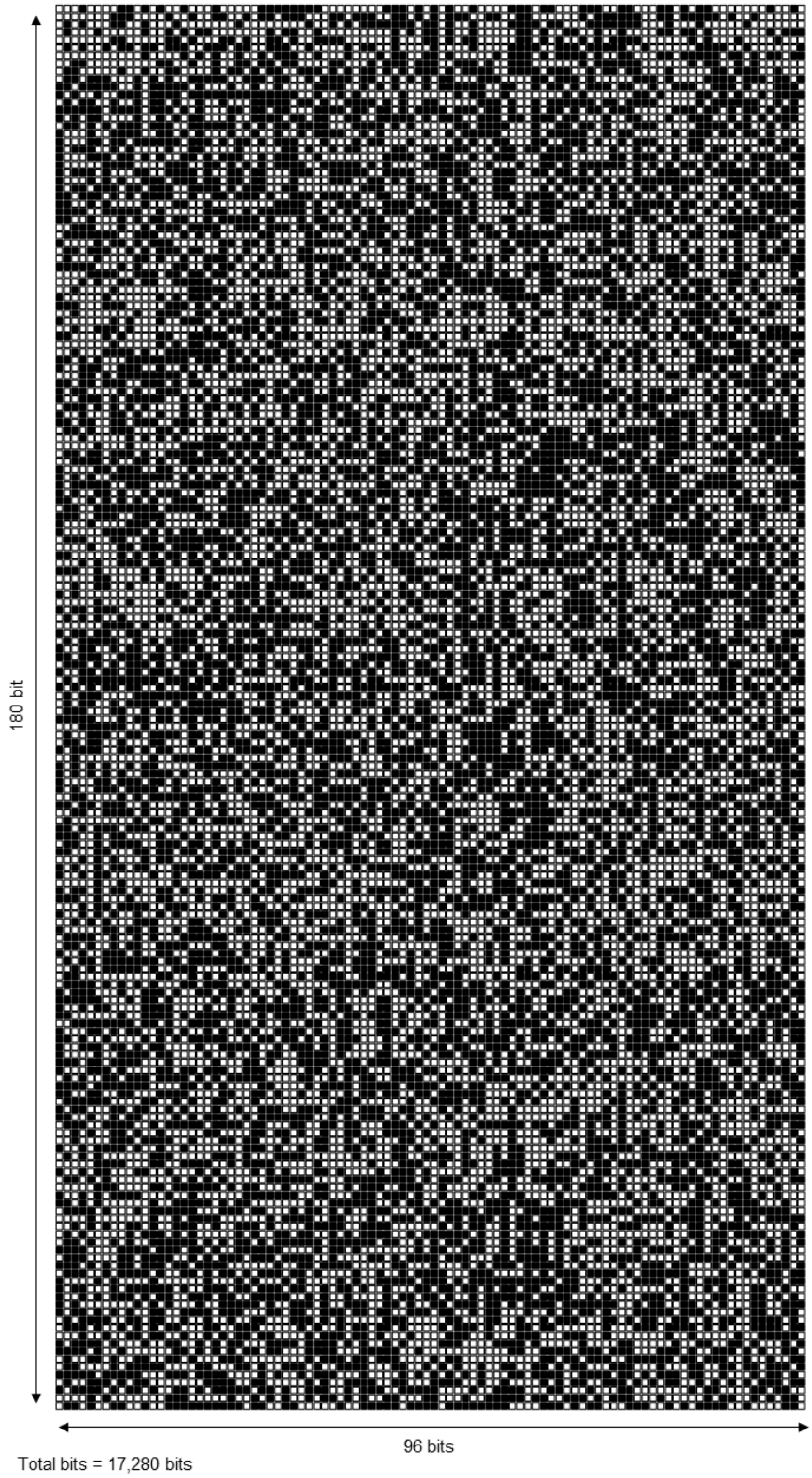

Supplementary Fig. 20 | Bitmap extracted for NIST randomness test. Bitmap extracted from 30 silk ID cards via a LOP-PUF module through nine challenge-response pairs (*i.e.*, Red +15°, Green +15°, Blue +15°, Red 0°, Green 0°, Blue 0°, Red −15°, Green −15°, and Blue −15°). A stream of 64 bits was generated by each challenge-response pair. As a result, a total of 17,280 bits were collected for a NIST randomness test.

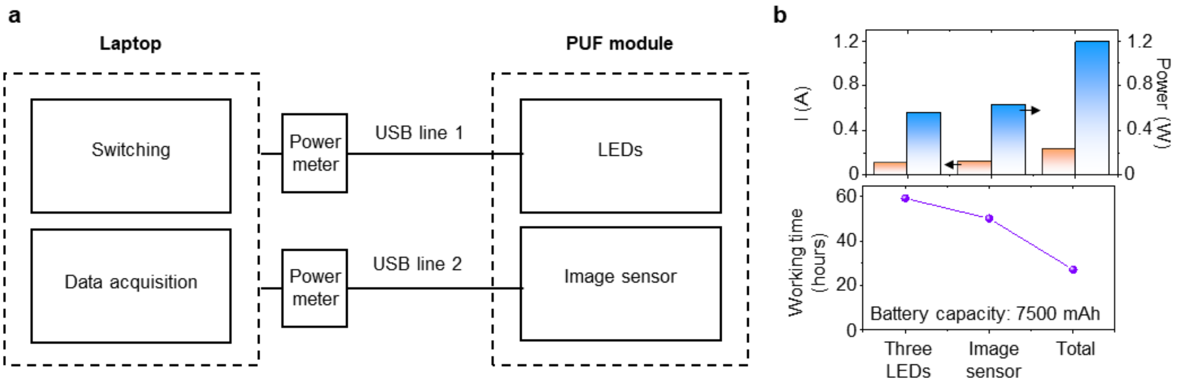

Supplementary Fig. 21 | Power consumption of LOP-PUF (a) Schematic for power consumption measurement of the LOP-PUF. Two USB-type power meters were used to measure the power consumption. (b) (top) Measured current and power and (bottom) working time of LOP-PUF operated by a portable battery with the capacity of 7500 mAh. The operating voltage is set to 5V.

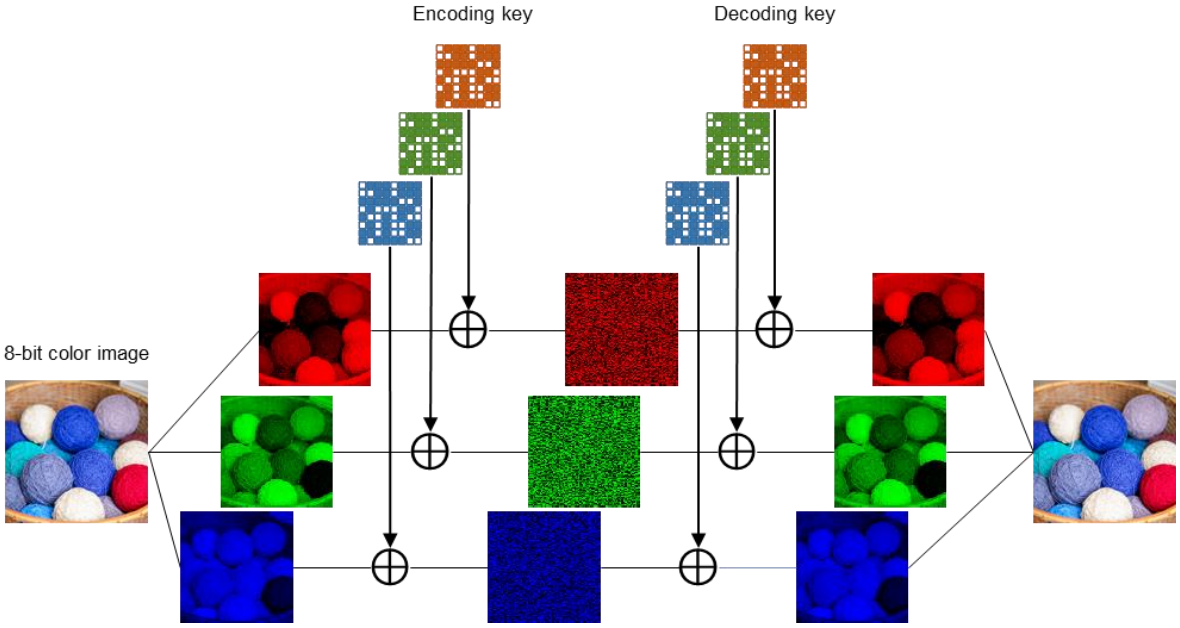

Supplementary Fig. 22 | Illustration of the data encoding and decoding system. The original data consisted of three-color space. For encoding and decoding, an XOR operation was performed on each channel data with the extracted PUF key data.

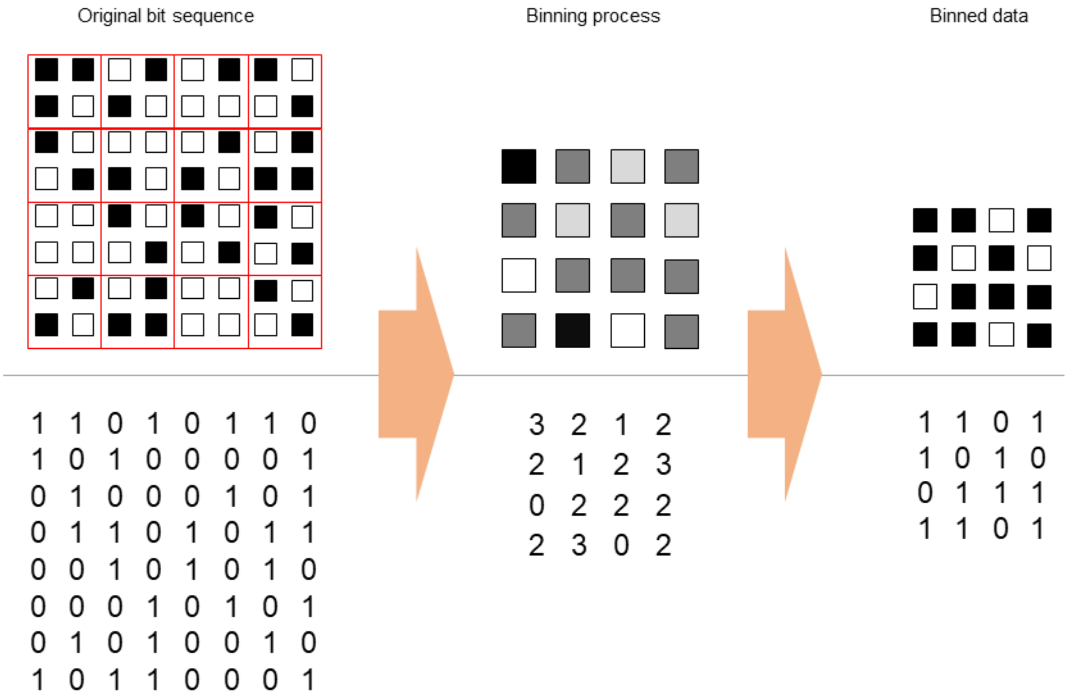

Supplementary Fig. 23 | Process of data binning. An illustration of data binning. The original data has noise bit elements due to the edges of the peak point. Binning was performed to cluster the bits into a unit of a predetermined dimension so that the noise at the edge had a smaller influence than the peak signal.

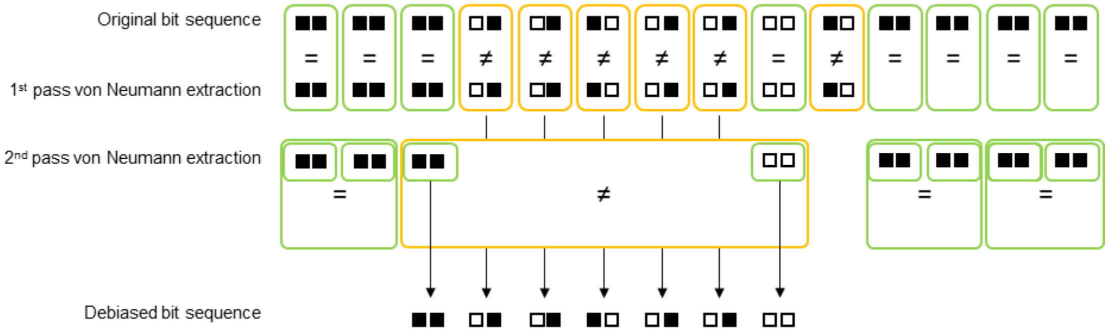

Supplementary Fig. 24 | Process of von Neumann debiasing. An illustration of von Neumann debiasing. The extracted bit sequence was biased at ‘0’ because the number of peak points was smaller than whole domain. Thus, 2 pass von Neumann debiasing extraction was used to increase the uniformity of the bit sequence.

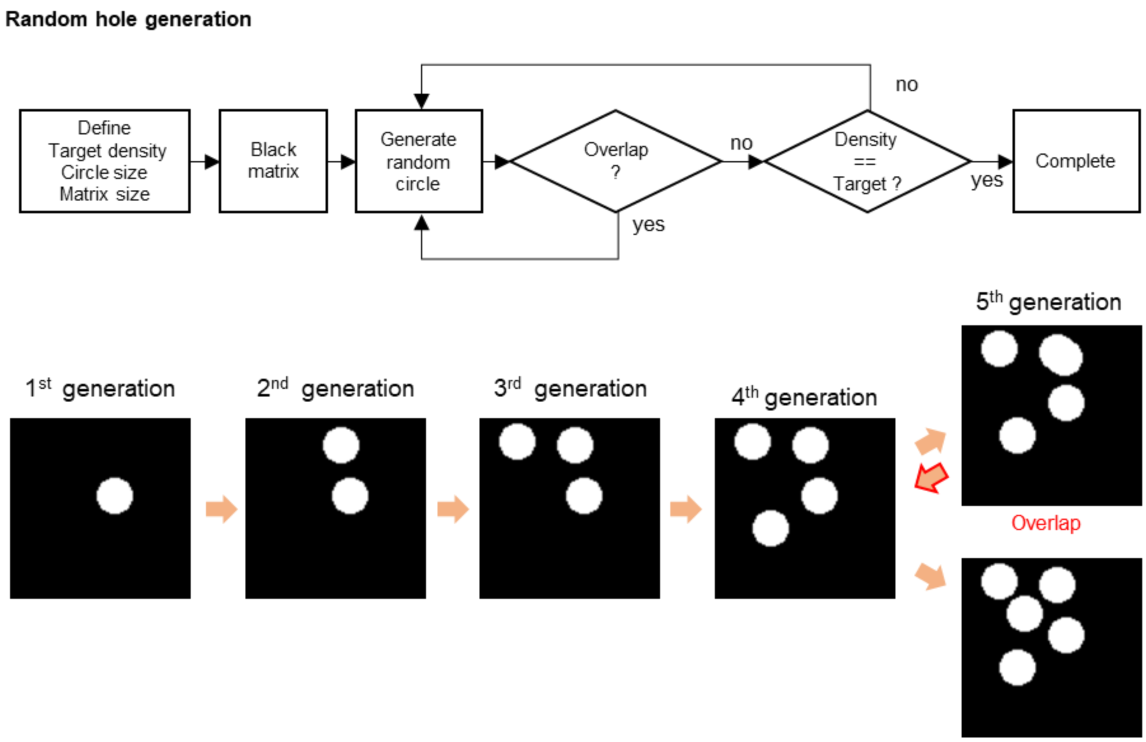

Supplementary Fig. 25 | Generation of random nanofibrillar structures. An illustration of the generation of random nanofibrillar structures in a single microfiber. A flow chart of random fiber generation (top) and a typical example (bottom). To confirm the density of the random fibers, the ratio of white and black areas was checked at every generation. Also, feedback was conducted to avoid any overlapping of the random holes.

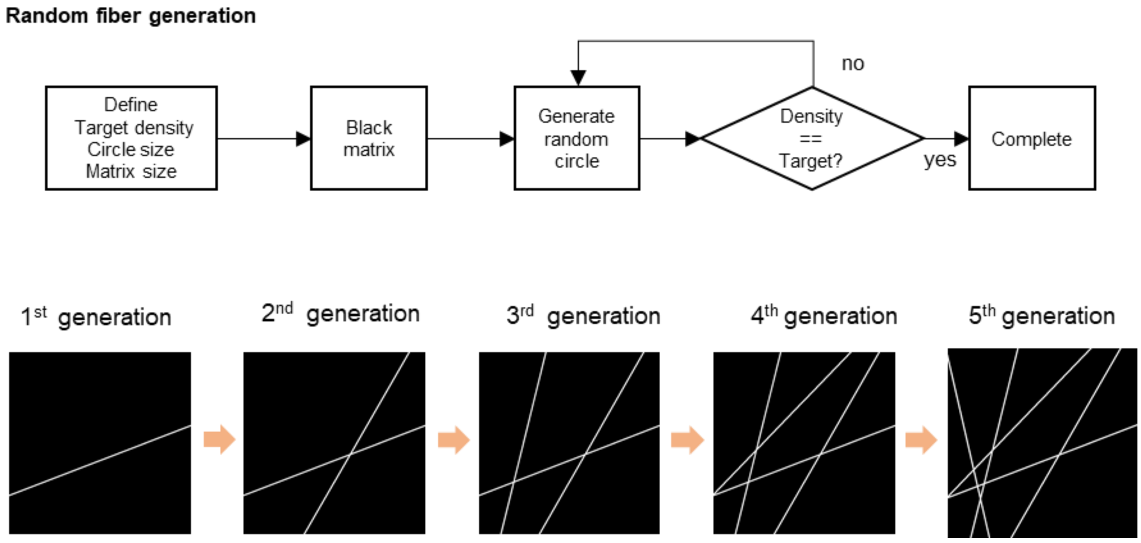

Supplementary Fig. 26 | Generation of random fibrous medium. An illustration of the random fibrous medium generation. A flow chart of the random fiber bundle generation (top) and a typical example (bottom). To confirm the density of the random fibrous medium, the ratio of the white and black areas was checked at every generation.

References

1. Wali, A. *et al.* Biological physically unclonable function. *Commun. Phys.* **2**, 39 (2019). doi:10.1038/s42005-019-0139-3.

2. Arppe-Tabbara, R., Tabbara, M. and Sørensen, T. J. Versatile and Validated Optical Authentication System Based on Physical Unclonable Functions. *ACS Appl. Mater. Interfaces* **11**, 6475-6482 (2019). doi:10.1021/acsami.8b17403.

3. Liu, Y. *et al.* Inkjet-printed unclonable quantum dot fluorescent anti-counterfeiting labels with artificial intelligence authentication. *Nat. Commun.* **10**, 2409 (2019). doi:10.1038/s41467-019-10406-7.

4. Smith, A. F., Patton, P. & Skrabalak, S. E. Plasmonic Nanoparticles as a Physically Unclonable Function for Responsive Anti-Counterfeit Nanofingerprints. *Adv. Funct. Mater.* **26**, 1315–1321 (2016). doi:10.1002/adfm.201503989.

5. Wigger, B., Meissner, T., Förste, A., Jetter, V. & Zimmermann, A. Using unique surface patterns of injection moulded plastic components as an image based Physical Unclonable Function for secure component identification. Sci. Rep. **8**, 1–9 (2018). doi:10.1038/s41598-018-22876-8.
